# Localization and function of Kinesin-5-like proteins during assembly and maintenance of mitotic spindles in *Silvetia compressa*

**DOI:** 10.1186/1756-0500-2-106

**Published:** 2009-06-15

**Authors:** Nick T Peters, Anne Catherine Miller, Darryl L Kropf

**Affiliations:** 1 Biology Department, University of Utah, Salt Lake City, Utah, USA

## Abstract

**Background:**

Kinesin-5 (Eg-5) motor proteins are essential for maintenance of spindle bipolarity in animals. The roles of Kinesin-5 proteins in other systems, such as Arabidopsis, Dictyostelium, and sea urchin are more varied. We are studying Kinesin-5-like proteins during early development in the brown alga *Silvetia compressa*. Previously, this motor was shown to be needed to assemble a bipolar spindle, similar to animals. This report builds on those findings by investigating the localization of the motor and probing its function in spindle maintenance.

**Findings:**

Anti-Eg5 antibodies were used to investigate localization of Kinesin-5-like proteins in brown algal zygotes. In interphase zygotes, localization was predominantly within the nucleus. As zygotes entered mitosis, these motor proteins strongly associated with spindle poles and, to a lesser degree, with the polar microtubule arrays and the spindle midzone. In order to address whether Kinesin-5-like proteins are required to maintain spindle bipolarity, we applied monastrol to synchronized zygotes containing bipolar spindles. Monastrol is a cell-permeable chemical inhibitor of the Kinesin-5 class of molecular motors. We found that inhibition of motor function in pre-formed spindles induced the formation of multipolar spindles and short bipolar spindles.

**Conclusion:**

Based upon these localization and inhibitor studies, we conclude that Kinesin-5-like motors in brown algae are more similar to the motors of animals than those of plants or protists. However, Kinesin-5-like proteins in *S. compressa *serve novel roles in spindle formation and maintenance not observed in animals.

## Background

Kinesins are a diverse group of molecular motors present in protozoans, fungi, plants, and metazoans [1]. They share a globular motor domain that hydrolyses ATP to facilitate movement towards the plus or minus end of microtubules [2]. Kinesins participate in structural organization and/or stabilization of microtubules and also transport cargo throughout the cytoplasm utilizing microtubules as molecular highways [2]. The Kinesin-5 group of the kinesin superfamily consists of plus-end directed homotetramers with two motor domains on each end [1]. They have been shown to function in spindle organization during mitosis in animal cells, remaining inactive and sequestered within the nucleus during interphase [1]. Specifically, Kinesin-5 motors are thought to function at the spindle midzone and maintain spindle bipolarity by walking towards the plus ends of interdigitating microtubules from opposite poles [3]. Kinesin-5 motors are also present at spindle poles where they may create an outward force on parallel microtubules [4].

Monastrol is a cell permeant inhibitor of Kinesin-5 motors and is thought to function by binding the motor domains, thereby blocking normal movement [5,6]. Specifically, monastrol has been shown to bind the Kinesin-5-ADP complex and inhibit ADP release and may inhibit motor binding to microtubules [7,8]. Therefore, monastrol is a powerful tool to probe the functions of Kinesin-5 motors without the necessity of genetic manipulations. In animal cells, monastrol treatment induces spindles to collapse to monasters, while having no detectable effects during interphase [9]. In contrast, other organisms such as Dictyostelium and vascular plant cells appear to be monastrol insensitive [10,11]. Thus, sensitivity to monastrol appears to vary between different lineages.

It has previously been shown that monastrol treatment leads to malformed spindles in brown algae. Monastrol treatment of *Silvetia compressa *zygotes prior to mitosis induced the formation of mostly multipolar spindles; monasters were also formed albeit to a lesser degree [12]. Monastrol treatment also induced formation of numerous cytasters during mitosis, likely due to spindle pole fragmentation, but did not affect interphase microtubule arrays. These findings suggested that brown algal Kinesin-5-like motors, like animal Kinesin-5 motors, are sensitive to monastrol treatment. However, the specific localizations and functions of Kinesin-5-like motors remain unclear in the brown algal lineage.

Here we examine Kinesin-5-like localization during early embryonic development and utilize monastrol to assess motor function in maintaining spindle structure and function in *S. compressa *zygotes. We show that Kinesin-5-like motors are localized within the nucleus during interphase, and strongly localize to the spindle poles from the onset of mitosis until nuclear envelope reformation. These motors are also associated with polar microtubules and the spindle midzone but to a much lesser degree. Additionally, we show that inhibition of Kinesin-5-like motors during mitosis leads to the formation of multipolar and short bipolar spindles, indicating that motor function is needed to maintain spindle integrity. Many of our findings are in concert with those found in animal cells. In both brown algal zygotes and animal cells, Kinesin-5 proteins are localized within the nuclear envelope until mitosis where they strongly decorate the spindle poles, and monastrol treatment induces formation of aberrant spindles.

## Methods

**Figure 1:**
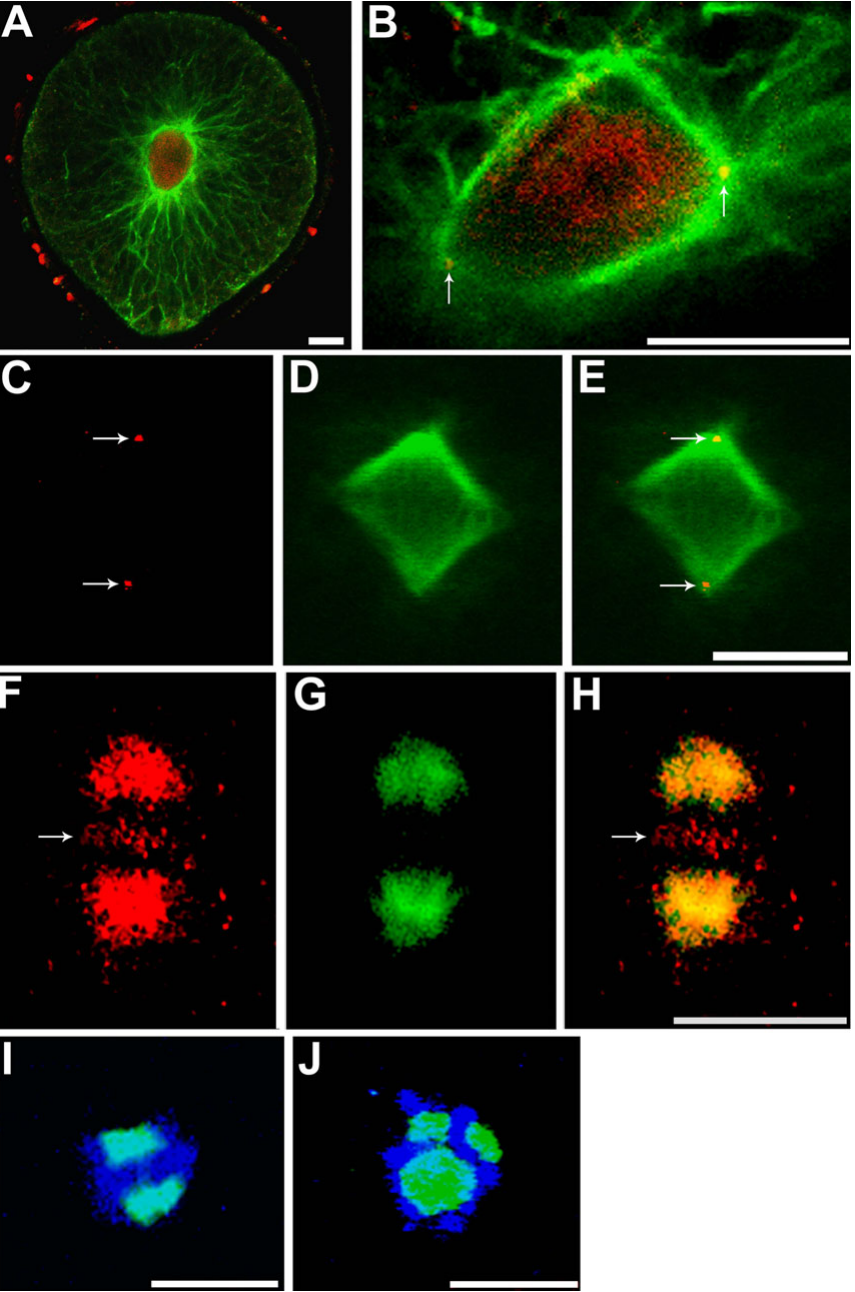
**Localization of Kinesin-5-like proteins (A-H) and morphology of short bipolar and multipolar spindles (I, J)**. Microtubules are in green in all images. Kinesin-5-like proteins are in red in A-C, E, F, H. Condensed chromatin is in blue in I and J. (A) 17-h old zygote in interphase. (B) Thallus cell of a 2-day old embryo entering prophase. (C-H) Two metaphase spindles are shown; anti-Kinesin-5 signal (C, F), microtubules (D, G), and merged images (E, H). In F and H, anti-Kinesin-5 signal was collected with increased amplifier gain and adjusted for contrast and brightness to enhance visualization of the weak signal; microtubule signal was not enhanced. Arrows indicate Kinesin-5-like motors colocalizing with microtubules in B and E, at the spindles poles and midzone in C and F, respectively. (I, J) Effect of monastrol treatment on metaphase spindles. Condensed chromatin is associated with short bipolar spindles in I and multipolar spindles in J following treatment with monastrol. Secondary antibodies commonly stick to zygotes' extracellular adhesive and is seen in A. Scale bars equal 10 μm.

Zygotes were cultured as previously described [12]. Monastrol (AG Scientific, San Diego, CA) was dissolved in dimethyl sulfoxide to create a stock solution of 100 mM. 1-butanol (Sigma-Aldrich, St. Louis, MO) was applied directly into the aqueous culture medium at a concentration of 0.2%. The use of 1-butanol as a cell cycle synchronizing tool has been described elsewhere [13]. For immunofluorescence microscopy of Kinesin-5 motors, microtubules, or condensed chromatin, zygotes were fixed in PHEM (60 mM piperazine-N, N0-bis(2-ethanesulfonic acid), 25 mM HEPES, 10 mM EGTA, 2 mM MgCl_2_, pH adjusted to 7.5 with KOH) containing 3% paraformaldehyde and 0.5% glutaraldehyde. Fixed zygotes were processed as previously described [12]. Kinesin-5 motors were labeled with polyclonal anti-Eg5 peptide primary antibody (Novus Biologicals, Littleton, CO) and Alexa 546 goat anti-rabbit secondary antibody (Invitrogen, Eugene, OR). Microtubules were labeled with a monoclonal anti-α-tubulin (DM1A) primary antibody (Sigma-Aldrich, St. Louis, MO) and Alexa 488 goat anti-mouse secondary antibody (Invitrogen, Eugene, OR). Condensed chromatin was labeled with a polyclonal anti-histone H3 (pSer10) antibody (Calbiochem, San Diego, CA), followed by Alexa 546 goat anti-rabbit secondary antibody. For double labeling of microtubules and either Kinesin-5 or condensed chromatin, primary antibodies were added simultaneously, as were secondary antibodies. Images were collected on an LSM510 (Carl Zeiss Inc., Thornwood, NY) confocal laser scanning microscope using a narrow bandpass filter for microtubules (500–530 nm) and a Meta adjustable bandpass filter (558–601 nm) for Kinesin-5 or condensed chromatin. Images were adjusted for brightness and contrast, and images shown in Fig. 1F–H were taken with increased amplifier gain to exaggerate anti-Kinesin-5 signal. All images represent single confocal sections of approximately 0.5 μm thickness. All experiments were repeated in triplicate and each data point represents a sample size of at least one hundred.

## Results and Discussion

### Kinesin-5-like proteins are localized to mitotic spindles

An anti-Eg5 polyclonal antibody directed towards the coiled coil domain of human Eg5 was used to establish the localization pattern of putative Kinesin-5 proteins in *S. compressa*. During interphase of the first cell cycle, anti-Kinesin-5 signal was observed exclusively within the nucleus (Fig. 1A). In prophase labeling was distributed throughout the elongated nucleus, and also distinctly localized to the forming spindle poles (Fig. 1B). During metaphase, localization was mainly observed at spindle poles (Fig. 1C, E, F, H) but weak signal was also occasionally observed at the spindle midzone in images taken with increased amplifier gain (Fig. 1F, H). Note that in Fig. 1F–H the spindle poles are out of the focal plane, allowing for better visualization of faint anti-Kinesin-5-positive signal at the spindle midzone.

The subcellular localization of Kinesin-5-like motors described above is generally similar to localization patterns observed in animals [14,15]. We interpret this localization pattern to be *bona fide *for several reasons. First, a tBLASTn search of the related brown alga *E. siliculosis *genome database [16] with full length human Kinesin-5 (KIF11) produced a match of 37% identity and 51% similarity across 239 amino acid residues, including half of the human KIF11 coiled-coil domain to which the antibody was made. Second, the *S. compressa *Kinesin-5-like motor is likely to be structurally and functionally similar to the human motor protein since both are sensitive to monastrol. Third, while anti-Kinesin-5-positive signal is seen most heavily at the forming spindle poles during prophase and at metaphase spindle poles, it is never observed to colocalize with centrosomes in interphase cells. This suggests that the strong association with the spindle poles is not due to non-specific centrosomal labeling. Finally, the weak labeling at the spindle midzone is consistent with the monastrol-induced spindle abnormalities described below. While these observations do not prove that the Kinesin-5 antibody exclusively binds to Kinesin-5 proteins, they strongly suggest it.

#### Disruption of Kinesin-5-like proteins leads to aberrant spindle morphology

**Figure 2:**
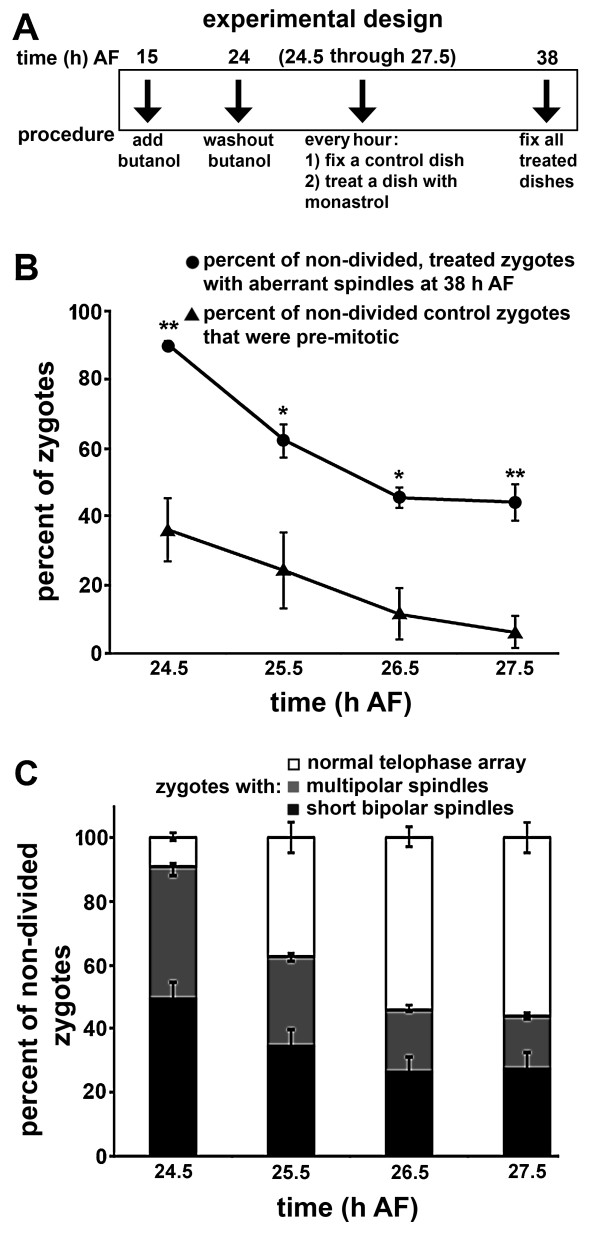
**Developmental progression of spindle formation and spindle morphology**. (A) Experimental design. Zygotes were treated with 1-butanol at 15 h AF and released by washout at 24 h AF. At hourly intervals from 24.5 to 27.5 h AF, a control dish was fixed and another dish was treated with monastrol. At 38 h AF, the monastrol-treated zygotes were fixed and labeled. (B) Percent of butanol-synchronized control zygotes that had not yet formed spindles (triangles) at the indicated times, and the percent of monastrol-treated zygotes with aberrant spindles at 38 h AF (circles). Cells that had divided were not included in this analysis. Control and treated populations were significantly different at all time points (single asterisk indicates P < 0.05 and double asterisks indicate P < 0.01). (C) Percent of telophase, short bipolar and multipolar spindles in monastrol-treated zygotes. The vast majority of the zygotes in telophase divided normally by 48 h AF. Standard error bars are shown.

Previous findings showed that addition of monastrol prior to entry into mitosis resulted in multipolar spindles and numerous cytasters, indicating that the putative Kinesin-5 motor is needed for proper spindle assembly [12]. In order to determine whether Kinesin-5-like proteins also function to maintain bipolarity in already established spindles, populations of zygotes were synchronized during mitosis (Fig. 2A). This was accomplished by application of 1-butanol, which reversibly arrests zygotes in metaphase with condensed chromatin and unseparated spindle poles [13]. 1-butanol was applied to zygotes prior to mitotic entry (15 h after fertilization) and by 24 h after fertilization nearly all zygotes had arrested in metaphase (data not shown). Zygotes were released from 1-butanol arrest by washout from 24 to 24.5 h after fertilization, and control zygotes were fixed at hourly intervals thereafter to assess spindle maturation. Simultaneously, another sample was treated with monastrol; the later the time of monastrol addition the more mature the spindles were at the time of treatment. The monastrol-treated zygotes were allowed to develop until 38 h after fertilization and were then fixed and labeled to analyze spindle morphologies. Interestingly, attempts to label Kinesin-5-like proteins in monastrol-treated zygotes were unsuccessful; perhaps due to reduced affinity of Kinesin-5-like proteins for microtubules in the presence of monastrol [8].

Regardless of the time of application, monastrol treatment led to the formation of very short, but still bipolar, spindles and also to multipolar spindles (Fig. 1I and 1J). Multipolar spindles have also been observed following Kinesin-5 gene disruption in higher plants and in invertebrate cells [10,14]. Cytasters were infrequently found in cells possessing short bipolar spindles. The maximal pole-to-pole distances of monastrol-treated and control spindles were measured. Short bipolar spindles were approximately half the length of untreated control spindles (Table 1). In contrast to animals [9], spindles of *S. compressa *rarely collapsed fully to monasters.

**Table 1:** Spindle pole-to-pole distance in control and monastrol-treated zygotes

Spindle category	Distance (μm) ± SE
Washout control spindles	17.39 ± 0.12
Short bipolar spindles in monastrol-treated zygotes	7.75 ± 0.44^†^
Multipolar spindles in monastrol-treated zygotes*	19.22 ± 0.24

† indicates significant (P < 0.01) difference from controls or multipolar spindles

* greatest distance between two spindle poles

Following 1-butanol washout at 24 h after fertilization, control zygotes entered mitosis very rapidly; most had formed spindles within 30 min. The percent of zygotes that were pre-mitotic dropped from 36.6 ± 9.3% at 30 min after washout to 6.6 ± 4.8% at 3.5 h after washout (Fig. 2B). The percent of aberrant (short bipolar plus multipolar) spindles in the monastrol-treated zygotes was significantly higher than the percent of zygotes containing pre-mitotic spindles at the time of drug addition (Fig. 2B). Thus, the aberrant spindles must not arise exclusively from zygotes entering mitosis and improperly assembling a spindle. That is, a large portion of disrupted spindles must have originated from pre-formed bipolar spindles that partially collapsed or broke apart. This suggests that in brown algae, Kinesin-5-like proteins are required for maintenance of spindle integrity.

Spindle morphology and progression through the cell cycle were analyzed in greater detail in the monastrol-treated populations. As expected, the later monastrol was added, the greater the percentage of zygotes that progressed to telophase by 38 h after fertilization (Fig. 2C), suggesting these zygotes were past metaphase when drug was applied. Thus, the percentage of aberrant spindles decreased with later treatment time. The percentage of zygotes with multipolar spindles declined more steeply with time than did the percentage with short bipolar spindles.

These findings suggest that monastrol treatment may have different effects on cells entering mitosis compared to those that already possess a spindle. We previously reported that treatment of young zygotes with monastrol well before entry into mitosis resulted in nearly all cells forming multipolar spindles [12]. Thus, spindle pole breakup and formation of multipolar spindles may preferentially occur as spindles are assembled. The current data are consistent with this hypothesis. The percentage of cells that were pre-mitotic at the time of drug addition was quite similar to the percentage of cells forming multipolar spindles following treatment. Furthermore, zygotes treated later in mitosis tended to form relatively more short spindles than multipolar spindles. An interesting possibility is that the formation of short spindles is due to shortening of bipolar spindles upon drug addition, whereas the formation of multipolar spindles is due to improper assembly of spindles, leading to spindle pole breakup prior to achievement of bipolarity. It does however remain a formal possibility that monastrol-treated spindles form properly but break apart or collapse while trying to maintain bipolarity.

## Discussion

Kinesin-5 motors are highly conserved throughout the eukaryote lineage [1]. Interestingly, while the localization and function of these motors in *S. compressa *and animals are similar, they are distinct from those of higher plants, protists, and invertebrates. In *S. compressa *and animals, Kinesin-5-like motors are sequestered within the nucleus during interphase and localize to the spindle poles during mitosis, but in higher plants these motors decorate cortical microtubules throughout the cell cycle [10]. Furthermore, disruption of motor function with monastrol induces spindle abnormalities in both *S. compressa *and animal cells, but has little effect on higher plant or Dictyostelium cells [9-12,17]. These observations suggest that the mechanisms by which brown algae build and maintain bipolar spindles are more closely related to mechanisms operating in animal cells than to those of vascular plants. While the extent of this relatedness has yet to be fully understood, it may be due in part to ancient conserved mechanisms of spindle formation possessed by centrosomal organisms but lacking in non-centrosomal ones. There is, however, a difference between the abnormal spindles formed in *S. compressa *and animal cells. Animal cells exclusively form monasters whereas the algal zygotes produce multipolar and short bipolar spindles, and to a lesser degree monasters. The lower incidence of monasters may indicate that in brown algae additional forces, perhaps provided by other motors, function to maintain some degree of spindle pole separation in the absence of Kinesin-5-like activity.

## Abbreviations

(AF): after fertilization; (ASW): artificial sea water.

## Competing interests

The authors declare that they have no competing interests.

## Authors' contributions

NTP and ACM carried out the experiments, NTP and DLK wrote the manuscript, and DLK raised the funds. All authors have read and approved the final manuscript.
